# Three‐Dimensional Ovary Model to Improve and Study Murine Follicle Growth

**DOI:** 10.1002/adhm.202504643

**Published:** 2026-02-24

**Authors:** Mira Jacobs, Valon Gllareva, Lukas Moser, Silvia Pravato, Eric Mora Pimentel, Brigitte Leeners, Martin Ehrbar

**Affiliations:** ^1^ Department of Obstetrics University Hospital Zurich University of Zurich Zurich Switzerland; ^2^ Department of Reproductive Endocrinology University Hospital Zurich University of Zurich Zurich Switzerland

**Keywords:** ECM secretion, in vitro maturation, mouse follicle culture, ovary model, Poly(ethylene glycol) hydrogel

## Abstract

To increase chances for women with impaired gonadal function to conceive biological offspring in vitro platforms for cultivating small follicles are developed. These rely mainly on the manual supplementation of required hormones and growth factors. We hypothesize that within a 3D ovary model, direct interactions between stroma cells and follicles would improve follicle viability and growth, thereby resulting in a larger pool of mature follicles. In this study, a transglutaminase crosslinked poly(ethylene glycol) (TG‐PEG) is adapted in its stiffness and functionalized with the cell adhesion peptide RGD to allow growth of follicles and to render it bioactive. During seven days of culture, the follicles grow, retain important morphological characteristics, and oocytes acquire meiotic competence. Following this, a co‐culture system that utilizes either paracrine signaling between follicles and supporting cells or a co‐culture that relies on direct contact is employed, confirming improved follicle growth when cultured with support cells. Confocal imaging reveals formed cell–cell interactions between support cells and follicles in detail. This newly developed ovary model consisting of a highly tunable TG‐PEG hydrogel, follicles, and support cells facilitates functional follicle growth in vitro and allows to study follicle‐stroma cell interactions to help improve fertility preservation techniques.

## Graphical abstract

To develop new methods for fertility preservation in women, novel culture systems for small follicles need to be established. In this work, a poly(ethylene glycol) based scaffold, with appropriate mechanical and bioactive features to support mouse follicle and ovary cell growth is developed, and the formed cell‐follicle interactions are visualized.

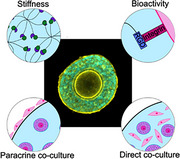

## 1 Introduction

Fertility preservation is the only option for female cancer patients to conceive children after emergent treatment with gonadotoxic cancer drugs or radiotherapy [1, 2, 3] as well as for women receiving medication with a severe negative impact on ovarian function. For some patients, this can be achieved by re‐implantation of cryopreserved autologous ovarian tissue that has been harvested before the onset of cancer therapy. To prevent the reintroduction of malignant cells with the transplanted tissue, for patients with hematologic malignancies, therapies must rely on isolated and cancer cell‐free mature follicles [4, 5]. Even in reproductive age, the human ovaries contain only a few large and growing follicles that are located in the inner part, the medulla [6, 7]. The majority of follicles are less developed primordial follicles, making up the ovarian reserve that reside in the stiff outer ovarian cortex. During the menstrual cycle, these follicles undergo a growth and maturation process regulated by the hypothalamus and pituitary gland, called folliculogenesis. In addition, follicular maturation depends on intricate juxtacrine and paracrine signaling among follicular cells, including the oocyte, granulosa, and theca cells, as well as the surrounding ovarian stroma [8, 9].

Ideally, fertility preservation methods would rely on the growth and maturation of the large pool of primordial and primary follicles [3, 5]. The ability to culture and treat such isolated follicles individually would increase the number of available mature follicles and therefore the likelihood for patients to produce biological offspring through in vitro fertilization (IVF). The successful in vitro growth and maturation of oocytes would open new opportunities to decrease health risks and financial burden. Additionally, it would strongly increase efficiency in fertility preservation for non‐medical reasons, which is currently one of the most growing fields in reproductive medicine. However, finding culture conditions that enable the generation of mature follicles from primordial ones has been challenging. The establishment of such conditions is complicated by the fact that follicles consist of multiple different cell‐types and must retain morphological characteristics such as gap junctions between granulosa cells and basal lamina integrity. Their disruption results in the loss of oocyte function due to insufficient metabolic exchange and granulosa cells migrating away from the oocyte, respectively [10, 11, 12, 13].

So far, protocols for the culture and maturation of follicles have been mostly established with murine follicles, due to the limited access to human follicles for research. It has been shown that murine follicles between approximately 110–180 µm in diameter can be grown and matured in vitro, such that oocytes can be fertilized [14, 15]. Promising results were reported when follicles were embedded and 3D cultured in biomaterials like the extracellular matrix (ECM) proteins collagen and fibrin [16, 17]. Compared to 2D cultures on plastic, this 3D‐system was superior at preserving the delicate follicle morphology, including the retention of the basal lamina and cell–cell connections between granulosa cells and the oocyte, crucial requirements for follicle development and function [18]. However, while these biomaterials provided ECM cues important for follicle development, they were mechanically and proteolytically unstable and degraded fast, both in vitro and in vivo. For example, in one study primordial follicles embedded in fibrin, developed into secondary follicles during 21 days of transplantation in the ovarian bursa of mice, but during this time the number of transplants decreased to 40% due to fibrin degradation [19].

Alginate, a natural biomaterial, is composed of blocks of guluronic and mannuronic acid, whereas the former, by coordination of divalent ions (usually Ca^2+^), enables instantaneous hydrogel formation [20]. Therefore, the stiffness can be tuned by the composition of the employed alginate monomers and by the concentration of divalent ions within the culture media [21]. Due to its easy crosslinking, optical transparency, and tunable stiffness, alginate is considered the state‐of‐the‐art biomaterial to encapsulate mouse follicles. However, in its natural form, alginate does not provide cell‐adhesion sites, nor can it be degraded by cell‐secreted enzymes. Additionally, in cell culture conditions, it can lose its stability and change mechanical properties due to leakage of Ca^2+^.

Therefore, to develop defined in vitro follicle culture systems, chemically crosslinkable synthetic poly(ethylene glycol) (PEG) hydrogels with tunable mechanical properties, proteolytic degradability, and presentation of cell adhesion sites, were introduced. Such PEG hydrogels were used to culture mouse follicles of size 100–120 µm and it was shown that follicle growth was significantly impacted by material stiffness [22]. Another study assessed PEG hydrogels that were functionalized with different ECM‐sequestering peptides to support follicle growth, significantly improving follicle survival, growth, and maturation compared to a bioinert control [23].

Despite these significant advances made with defined scaffold materials and media compositions, follicles likely require additional microenvironmental cues, such as the stromal juxtacrine or paracrine signals to more closely recapitulate the in vivo development. Thus, to support and boost follicle development with potential ovarian stromal signals a few investigations included additional cell types in their culture systems. For example, mouse embryonic fibroblasts were employed as a feeder layer to promote the growth of primary mouse follicles encapsulated in alginate‐based hydrogels through paracrine signaling [24, 25]. Similarly, another study proved that mouse follicle growth in alginate increased in the presence of a monolayer of ovarian stromal cells, consisting of mostly thecal cells and macrophages [26]. 3D co‐cultures were also established in PEG hydrogels using adipose derived stem cells and follicles to support follicle growth, as these cells have been shown to be effective in a range of human therapeutic settings. However, in this study, support cells were not of ovarian origin, they were added at low concentration (1 × 10^6^ cells ml^−1^ of hydrogel), and the hydrogel did not contain cell adhesion sites [27]. This suggests that 3D ovarian co‐cultures can be further optimized by systematically tuning individual parameters.

Here, we establish a culture system that enables the gradual recapitulation of cellular and structural properties of the natural ovarian stroma. For this, a biomaterial scaffold that is stable in cell culture conditions and is tunable to support the spreading and growth of embedded cells is required. We have previously developed a transglutaminase crosslinked PEG (TG‐PEG) hydrogel that thanks to its modular design fulfills these requirements optimally [28, 29, 30]. We have shown that TG‐PEG hydrogels serve as provisional substrates for the 3D culture of different stromal cell types, both in mono‐ and co‐culture [28, 29, 30, 31]. When adapting the stiffness, degradability, and presentation of adhesion sites of the material, embedded cells were able to spread, build networks, and secrete their own ECM.

In this study, to establish an engineered ovary model, we tailored the mechanical properties of TG‐PEG hydrogels for the culture of murine follicles at different developmental stages as these can be found in regions of different stiffness in the native ovary. Next, we rendered the scaffolds bioactive, by incorporating the cell adhesion peptide RGD. Finally, support cells were added into the culture system, and a paracrine and a direct co‐culture were compared in their follicle growth promoting effect. Such a system, besides improving follicle viability and growth provides a tunable platform to study and manipulate parameters that drive follicle growth and development.

## 2 Results

### 2.1 Formation and Mechanical Stability of TG‐PEG Hydrogels in Culture

**FIGURE 1:**
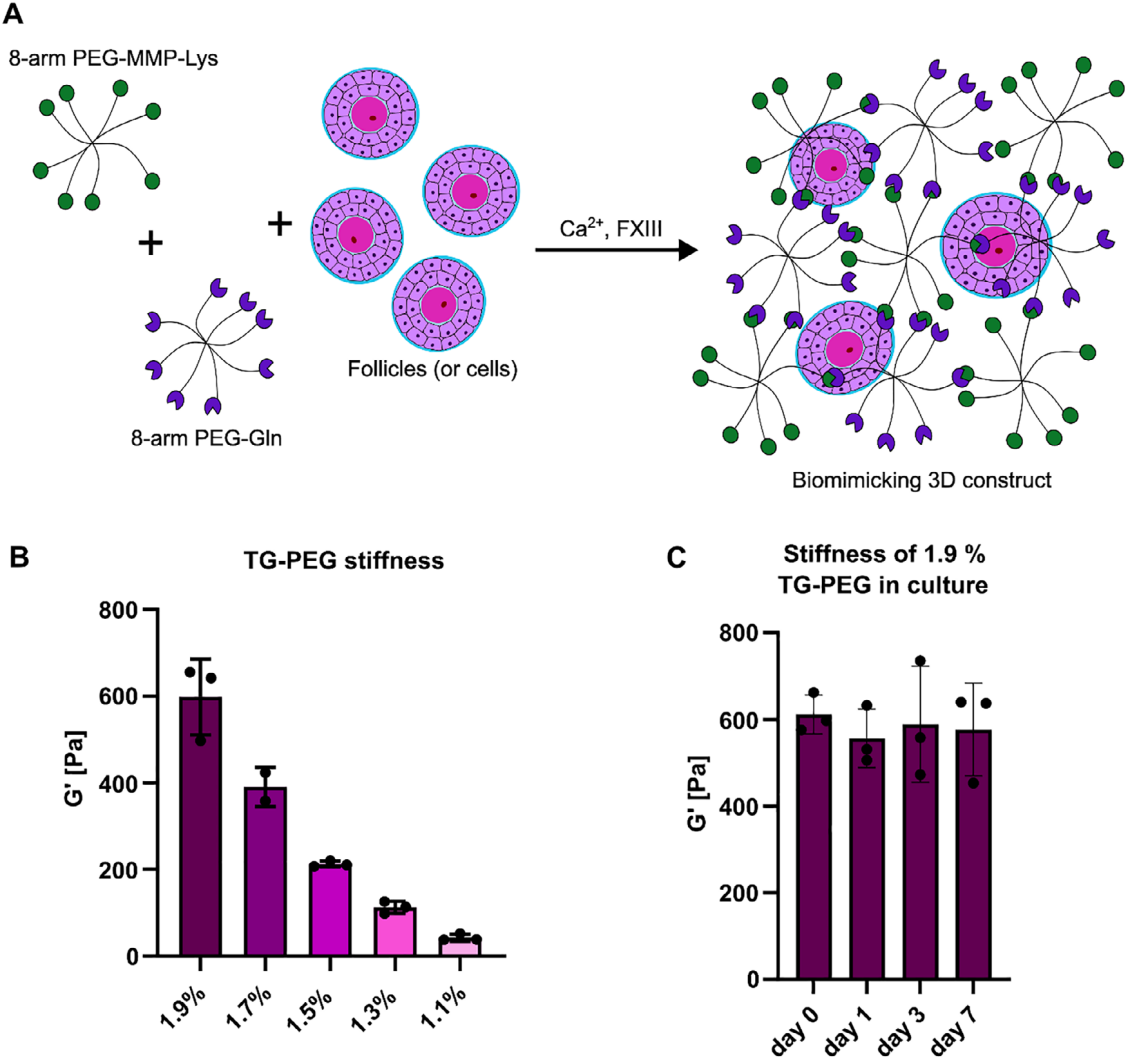
Formation of poly(ethylene glycol) hydrogels and mechanical properties. Schematic of hydrogel formation including the different components and cells. The two 8‐arm monomers are mixed with follicles or cells in a buffer containing calcium‐chloride and upon addition of the enzyme FXIII gelation starts (A). Stiffness of TG‐PEG hydrogels made from different monomer concentrations (B) and stability of stiffness under culture conditions (C). Stiffness depicted as storage modulus (G‘).

To build a 3D engineered ovary model, follicles and support cells were encapsulated in TG‐PEG hydrogels through the enzymatic cross‐linking of 8‐arm PEG monomers that were either functionalized with a glutamine‐donor (8‐arm PEG‐Gln) or a matrix metalloproteinase degradable lysine‐acceptor peptide (8‐arm PEG‐MMP‐Lys) (Figure 1A). To adapt their stiffnesses, first, the cell‐free hydrogels were formed with increasing concentrations (1.1%, 1.3%, 1.5%, 1.7%, and 1.9%) of PEG monomers. Oscillating rheological shear measurements showed that these hydrogels have stiffnesses of 50, 200, 400, and 600 Pa, respectively (Figure 1B). Furthermore, stiffness measurement of the hydrogels cultured for a week in follicle media at 37°C, 5% CO_2_ confirmed that the stiffness is stable during this time (Figure 1C).

### 2.2 Follicles Cultured in TG‐PEG Retain Important Morphological Characteristics

**FIGURE 2:**
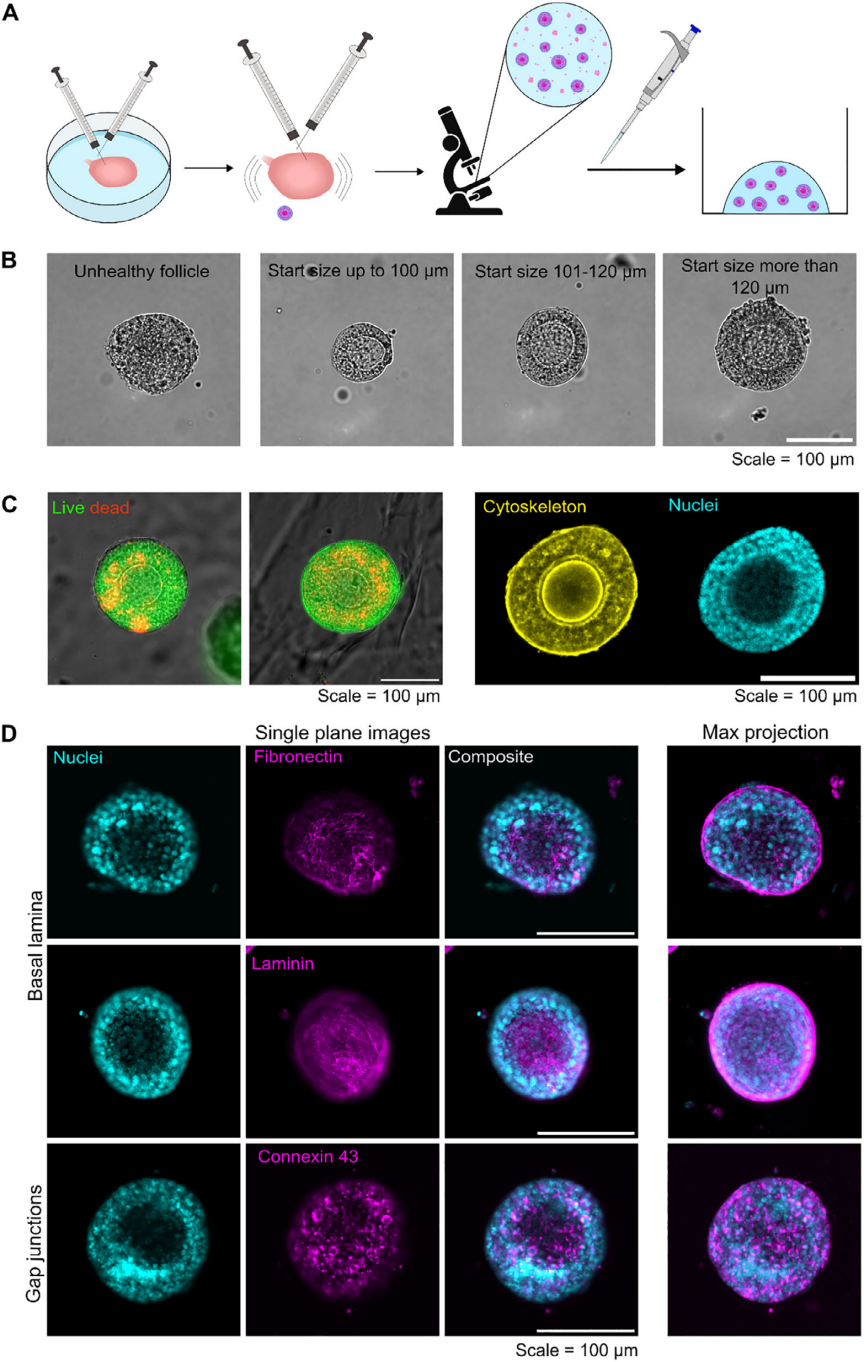
Mouse follicle isolation and 3D culture in TG‐PEG. Schematic of the isolation and encapsulation procedure (A). For growth analyses, follicles were classified from brightfield pictures into live and unhealthy. Living follicles were further classified into groups according to their starting size (B). Representative single plane pictures of follicles after seven days of culture, indicating viability and overall morphology of follicles. Dead cells stained with ethidium homodimer‐1 (in red) and live cells with calcein‐AM (in green). Follicle morphology can be assessed using phalloidin‐rhodamine to stain the cytoskeleton (in yellow) and DAPI to stain the nuclei (in cyan). For healthy follicles, a round centrally located oocyte can be detected from single plane confocal images (C). To further confirm follicle health, samples were stained for laminin, fibronectin, and a GC gap junction molecule connexin 43 (Cx43) (all shown in magenta) after culture. Cell nuclei are shown in cyan. Single plane confocal images depicting nuclei, and laminin, fibronectin, or Cx43 individually or in a composite, and maximum projections of acquired stacks shown as indicated (D). Scale = 100 µm.

Follicles were isolated from cleaned ovaries of 14–28 days old mice using a manual “flicking” technique and encapsulated in TG‐PEG hydrogels of intermediate stiffness (400 Pa) using a hanging drop technique (Figure 2A). Follicle growth was followed by longitudinal imaging at day 0, 3, 5, and 7 using a brightfield microscope and measuring two perpendicular diameters to calculate the mean diameter for each living follicle. For this, only intact, viable follicles, which were round in shape and had a visible, centrally located oocyte were used (Figure 2B). To distinguish between follicles of different sizes, they were classified into three different groups according to their size at day zero (start size): up to 100 µm, 101–120 µm, and more than 120 µm.

To assess if the TG‐PEG hydrogel is a suitable substrate for follicle culture, a live‐dead assay was conducted and showed the survival of most granulosa cells (GC) after seven days of culture (Figure 2C). Additionally, staining of nuclei and actin cytoskeleton with 4′,6‐diamidino‐2‐phenylindole (DAPI) and rhodamine‐phalloidin revealed a compact morphology and a round shape of the cultured follicle and oocytes. To reinforce these observations, the presence of an intact basal lamina and of gap junctions in GC, two key characteristics of healthy follicles, was investigated. Immune staining and confocal imaging showed the continuous presence of the basal lamina components fibronectin, and laminin around the whole follicle. Similarly, connexin 43 (Cx43) stained GC were evenly distributed throughout the GC layer, suggesting the functional integrity in the cultured follicles (Figure 2D).

### 2.3 Adjustment of TG‐PEG Hydrogel Stiffness for Mouse Follicle Culture

**FIGURE 3:**
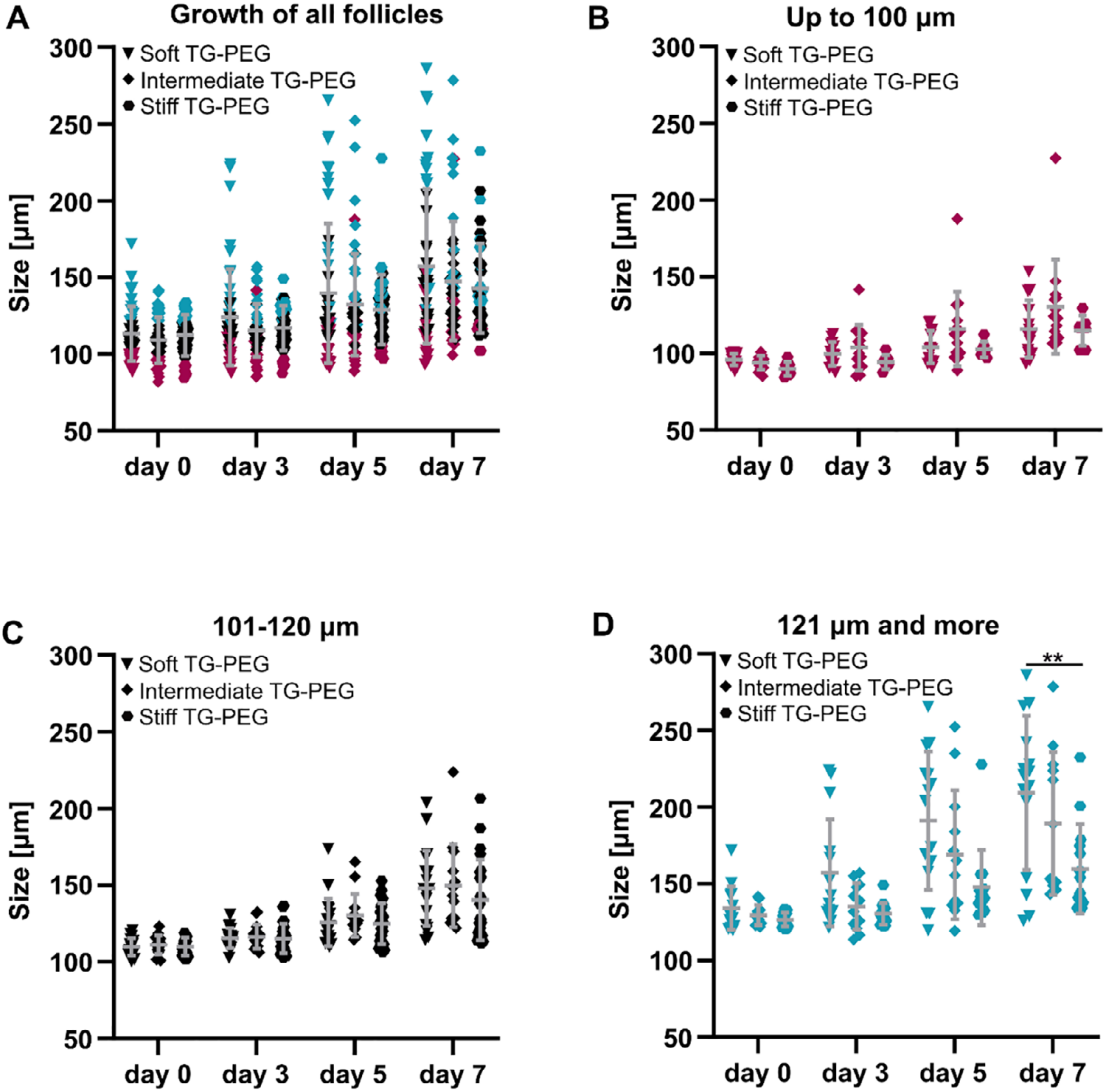
Growth analysis of follicles encapsulated in TG‐PEG of three stiffnesses. Follicles were embedded in soft, intermediate, and stiff TG‐PEG. Only data of follicles that were viable and growing during culture is plotted. Sizes from day 0, 3, 5, and 7 after encapsulation are shown for the different conditions (A), and for the different start sizes. Follicles up to 100 µm size (B), 101–120 µm (C), and more than 120 µm (D). Data of follicles of the smallest start size shown in red, medium size in black and large in blue. Data of growing follicles from N = 6 independent experiments and n = 22 ovaries. One‐way ANOVA with multiple comparisons used to determine statistical significances between conditions on day 7 (^**^ *p*<0.01).

To determine the effect of hydrogel stiffness on follicle growth, follicles were embedded in soft (50 Pa), intermediate (400 Pa), and stiff (600 Pa) TG‐PEG hydrogels. Between 79 and 111 follicles were encapsulated into hydrogels of each stiffness and the viability and growth were measured. No significant difference in follicle viability and number of growing follicles was observed, as 64%, 69%, and 61% of encapsulated follicles survived seven days of culture and 48%, 45% and 54% of the encapsulated follicles started to grow in soft, intermediate and stiff hydrogels, respectively (Table 1). Follicles embedded in the soft TG‐PEG hydrogels started to grow on average earlier than the ones embedded in stiffer hydrogels, and after seven days of culture, follicles in soft and intermediate TG‐PEG reached larger sizes than the ones cultured in the stiff hydrogels (Figure 3A). Otherwise, no clear effect of stiffness on follicle growth could be detected as the mean follicle size was not significantly different between groups after seven days of culture (Figure S1). To clearly see the effect of hydrogel stiffness on follicles of different developmental stages, growth data was plotted according to the start size of the follicles. The smallest follicles, which were up to 100 µm in size, grew to 130.4 ± 30.7 µm when embedded in intermediate TG‐PEG, and slightly less in the soft and stiff hydrogels (Figure 3B). Follicles of 101–120 µm start size grew on average to 147.9 ± 24.5 µm and 149.9 ± 27.1 µm in soft and intermediate TG‐PEG, respectively and slightly less to 140.3 ± 26.2 µm in the stiff hydrogel (Figure 3C). Follicles, which were largest upon encapsulation, grew to 209.3 ± 50.3 µm in the soft hydrogel, significantly more than in the stiff hydrogel, where they reached 129.6 ± 29.2 µm on average (Figure 3D; Figure S1). In intermediate TG‐PEG hydrogels they reached approximately 189.1 ± 46.6 µm in diameter.

**TABLE 1:** Numbers of encapsulated, surviving, and growing follicles for TG‐PEG hydrogels of three stiffnesses. After seven days of culture, follicles were identified as viable if there was a visible central round oocyte, intact and symmetrical appearing granulosa cells and an overall round follicle morphology detectable from brightfield images. Follicles were further classified as growing, if they increased in diameter compared to day 0. Values for surviving and growing follicles were assessed from the combined pool of all follicles per condition. This experiment was repeated in six independent experiments (N), and a total of 22 ovaries (n) were used.

7 days of culture in TG‐PEG	Soft	Intermediate	Stiff
# Encapsulated follicles	96	111	79
# Surviving follicles	61 (64%)	77 (69%)	48 (61%)
# Growing follicles	46 (48%)	50 (45%)	43 (54%)

Together, this shows that TG‐PEG stiffness does not significantly affect follicle growth of follicles up to 120 µm start size. However, the large follicles grow best in soft and intermediate TG‐PEG hydrogels. As the intermediate TG‐PEG hydrogels (400 Pa, 1.7% TG‐PEG) are easiest to handle in vitro and they allow for follicle growth, this stiffness was used for all following experiments.

### 2.4 Presence of RGD‐Peptide Enhances Follicle Growth

To mimic the natural ovary better, the follicle culture system should allow to co‐culture follicles and support cells in the same hydrogel. It was already shown that follicle survival and growth is enhanced if a 2D monolayer of support cells is present. A culture system, where both follicles and support cells are embedded together in 3D would better mimic the natural ovary. One requirement for cells embedded in 3D to secrete factors is spreading, and for this, the incorporation of an adhesion peptide is necessary when using a synthetic, bioinert hydrogel material like TG‐PEG.

**FIGURE 4:**
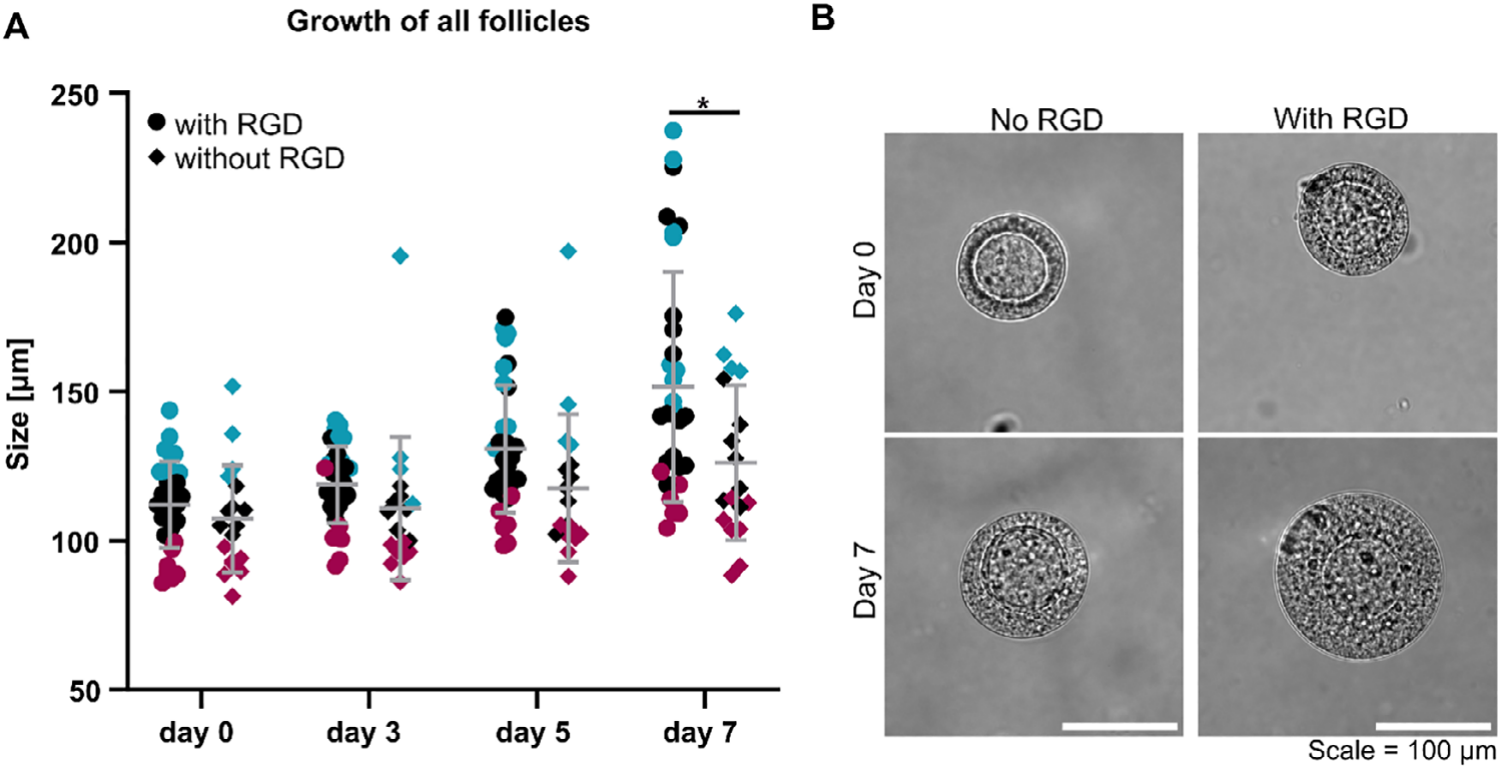
Growth data and morphology of follicles cultured in intermediate TG‐PEG hydrogels functionalized with or without RGD. Only data of follicles that were viable and growing during culture is plotted. Sizes from day 0, 3, 5, and 7 after encapsulation are shown. Data of follicles of the smallest start size shown in red, medium in black and large in blue. Data are from N = 3 independent experiments, and n = 8 ovaries. Unpaired t‐test was used to determine statistical significances between groups on day 7 (^*^ *p*<0.05) (A). Brightfield images taken at the end of the culture confirmed the integrity of follicles in the presence of RGD (B). Scale = 100 µm.

Comparison of follicles cultured in intermediate TG‐PEG functionalized with RGD or without showed that the viability of follicles was slightly higher with 76% in the presence of RGD compared to 61% without. The percentage of growing follicles was 56% and 50% (Table S1). Follicle size was significantly larger with RGD (151.6 ± 38.6 µm) compared to without RGD (126.2 ± 26.0 µm) (Figure 4A). Additionally, only with RGD follicles reached sizes of 200 µm and more after culture. When assessing follicles with different start sizes separately, the presence of RGD showed a non‐significant increase in growth (Figure S2). Longitudinal growth analysis revealed that follicles grew least within the first three days and most within the last two days of culture. When embedded with RGD, follicles grew between time‐points at least 1.5 times more compared to when no RGD was present (Table S3). To confirm that RGD is not leading to disintegration of follicles, follicle morphology was assessed. It was found that if the basal lamina was intact at the beginning of culture (clear and round follicle outline), the follicle maintained its round shape throughout the culture period (Figure 4B).

### 2.5 Follicles Grown in TG‐PEG Hydrogels Produce Oocytes with Acquired Meiotic Competence

**FIGURE 5:**
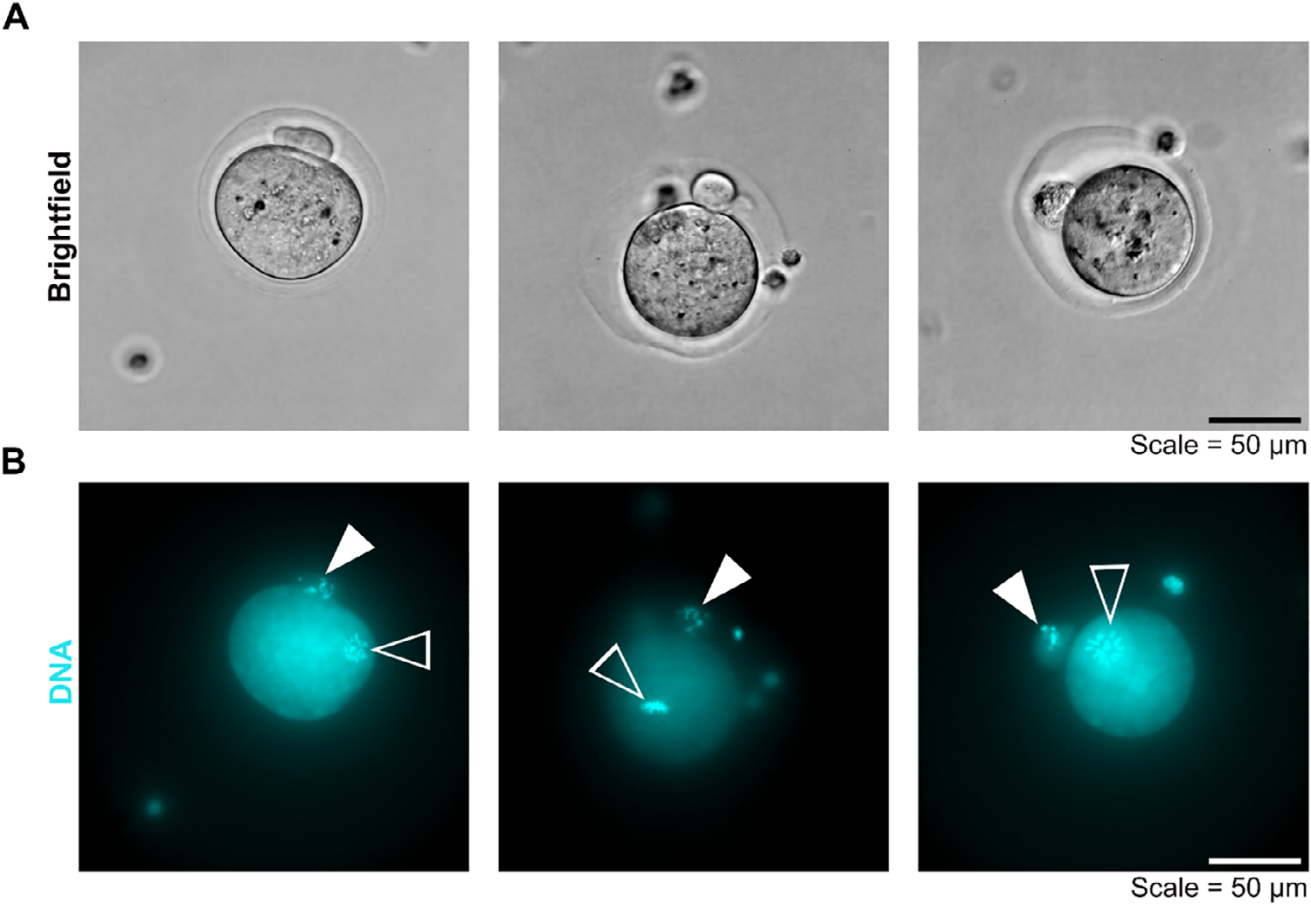
Representative images of oocytes in MII phase after culture in TG‐PEG‐RGD and IVM. Brightfield images of oocytes with an extruded polar body after seven days of culture and IVM (A). To confirm this, the DNA was stained in the same oocytes using Hoechst (in cyan). Single plane fluorescence microscope images are shown. Filled arrow heads point to the DNA within the polar bodies, empty arrowheads point to the DNA inside the oocytes (B). Scale = 50 µm. N = 1 independent experiments, and n = 2 ovaries.

To build an ovary model that supports growth and development of functional follicles GCs need to proliferate and the oocyte needs to mature for the egg to become fertilizable. Thus, an in vitro maturation (IVM) assay of follicles embedded in TG‐PEG‐RGD hydrogels for eight days was performed. Comparable to previous studies [14, 15], oocytes were able to enter metaphase II of meiosis, as seen by an extruded polar body, indicating that follicles cultured in transglutaminase crosslinked PEG hydrogels are able to produce oocytes with acquired meiotic competence (Figure 5).

### 2.6 Follicle Culture System with Support Cells Leads to Increased Follicle Growth

**FIGURE 6:**
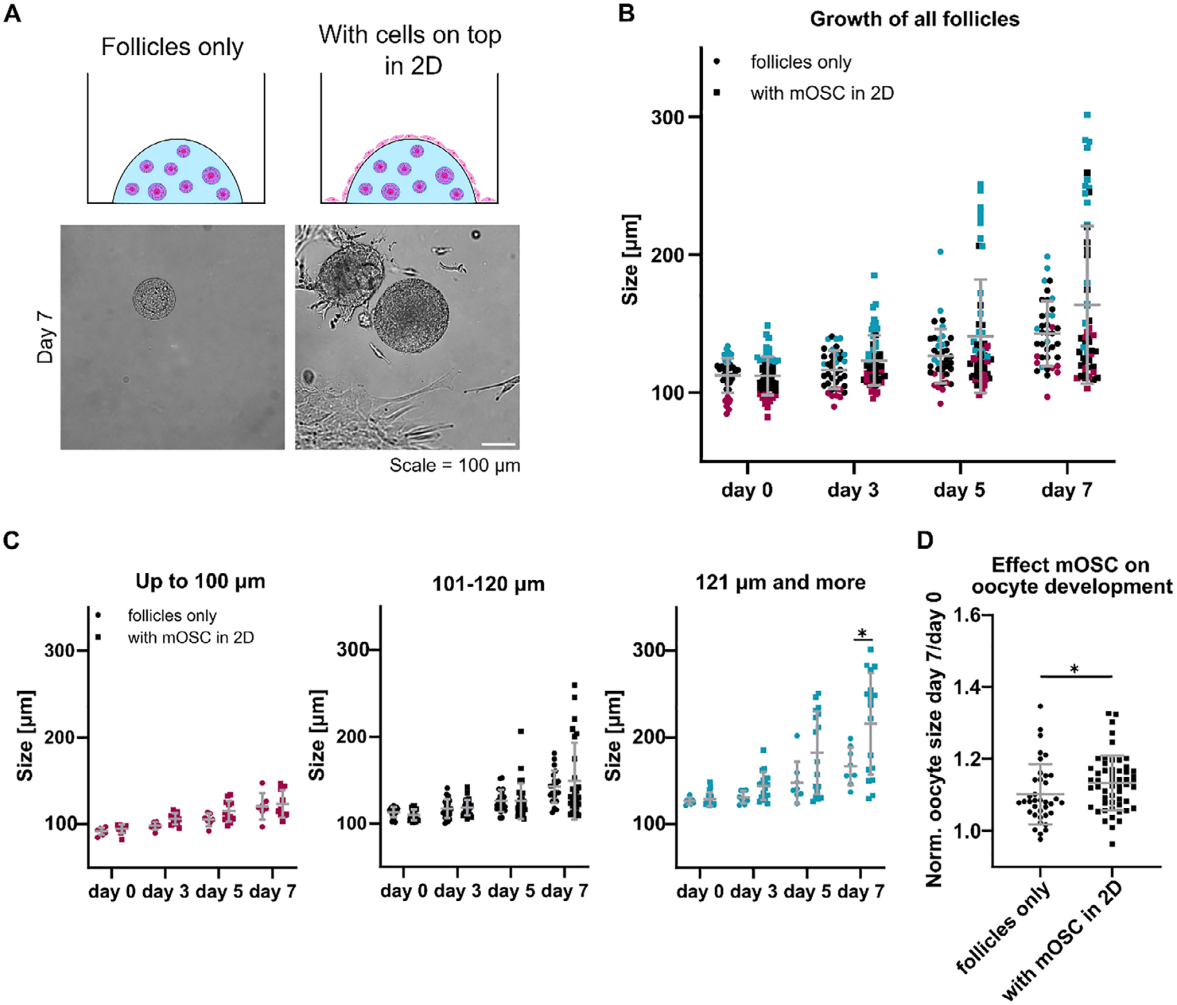
Growth data of follicles cultured in intermediate TG‐PEG‐RGD alone or with mouse ovary stroma cells. Scheme of the experimental conditions showing how follicles were either cultured alone in TG‐PEG‐RGD or with mOSC cultured in 2D on top of the hydrogels enabling paracrine signaling. Brightfield images show cell morphology after seven days of culture. Scale = 100 µm (A). Only data of follicles that were viable and growing during culture is plotted. Sizes from day 0, 3, 5, and 7 after encapsulation are shown. Follicles were embedded in intermediate TG‐PEG‐RGD either alone or with mOSC in 2D on top (B). Data of follicles per condition plotted and smallest start size are shown in red, medium in black and large in blue (C). Oocyte sizes from viable and growing follicles measured on day 0 and 7 of culture and the ratio day 7/ day 0 was plotted for each group (D). N = 2 independent experiments, and n = 8 ovaries. Unpaired t‐test was used to determine statistical differences between groups (^*^ *p* < 0.05).

To build a complete mouse ovary model and to improve currently available follicle culture systems a paracrine co‐culture was established with mouse ovary stroma cells (mOSC) as support cells. For this, similar to previously published approaches [25, 26], follicles were encapsulated in the hydrogels and support cells were seeded in 2D on top of the hydrogel. With this type of co‐culture, follicles get in contact with cell‐secreted factors, without direct physical contact to support cells (Figure 6A). Comparison of follicle growth when cultured alone or with mOSC on top of the hydrogels showed that the support cells enhance follicle growth during seven days, while they have no effect on viability (Figure 6B; Table S2). Especially, follicles with a start size larger than 120 µm grew significantly more when support cells were present (Figure 6C). Longitudinal evaluations revealed that follicles cultured in presence of mOSC, grew the least in the beginning and the most in the last two days of culture, comparable to follicle cultured without mOSCs. However, at all time‐intervals follicles cultured with mOSC grew at least 1.4 times more compared to follicles without (Table S3). To assess the effect of follicle co‐culture with mOSCs on oocyte development, oocyte sizes were determined. Comparable to follicle growth, oocytes grew significantly more when follicles were cultured in the presence of mOSC paracrine signals (Figure 6D).

### 2.7 3D Co‐Culture

**FIGURE 7:**
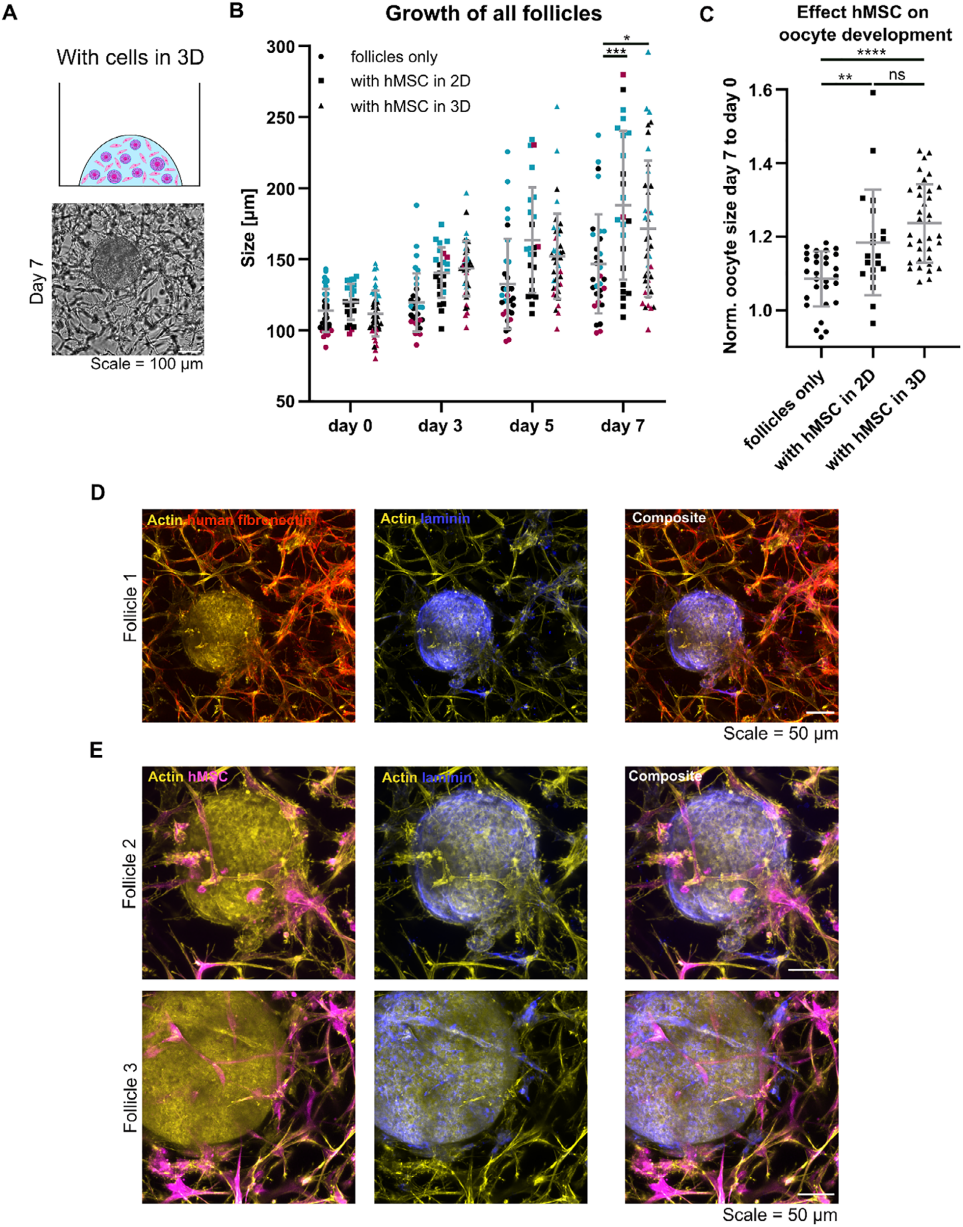
Growth data of follicles cultured in intermediate TG‐PEG‐RGD alone or with human mesenchymal stromal cells as model support cells. Scheme of the condition, where cells and follicles are embedded together. Brightfield image shows cell morphology after seven days of culture (A). Follicles were embedded for seven days in intermediate TG‐PEG‐RGD alone, with hMSC in 2D on top or together with hMSC in the same hydrogels. Only data of follicles that were viable and growing during culture is plotted. Sizes from day 0, 3, 5, and 7 after encapsulation are shown. Smallest start size are shown in red, medium in black and large in blue. (B). Oocyte sizes from viable and growing follicles measured on day 0 and 7 of culture and the ratio day 7/ day 0 were plotted for each group. N = 5 independent experiments, and n = 16 ovaries. One‐way ANOVA used to determine statistical significances between groups (^*^ *p*<0.05; ^**^ *p*<0.01; ^***^ *p*<0.001; ^****^ *p*<0.0001) (C). Immunofluorescence stainings of the samples after seven days of culture show ECM deposition of the different cell types as well as their organization in the co‐culture. Maximum projections of acquired confocal stacks (step size 10 µm) show either actin together with human fibronectin, actin together with laminin, or the composite of all three (D). Close‐up images depict the arrangement and points of contacts of hMSC, follicles, and their secreted matrix. Maximum projections of confocal stacks (step size 5 µm) show either actin together with hMSC, actin together with laminin, or the composite of all three (E). Actin of the cytoskeleton of all cells was stained with phalloidin‐rhodamine (in yellow), nuclei with DAPI (in cyan), and hMSC label with CellTrace Far Red (in magenta). Specific antibodies used for laminin (in blue) and human fibronectin (in red). Scale = 50 µm.

To evaluate if follicles can grow even more when they are encapsulated in the TG‐PEG hydrogel together with support cells, a direct co‐culture was performed such that cell–cell and cell‐matrix interactions can be established (Figure 7A). Since previous experiments showed that mOSC embedded in TG‐PEG hydrogels do not spread (unpublished data) we used human mesenchymal stromal cells (hMSC) as model support cells. These cells were previously shown to readily spread in TG‐PEG hydrogels while also secreting their own ECM [32].

A total of 67, 60, and 75 follicles were embedded in intermediate TG‐PEG‐RGD without support cells, with hMSCs in 2D on top of the hydrogel or together with hMSCs in 3D, respectively. From these follicles, between 43%–55% survived seven days of culture and 40%–55% started to grow (Table 2). Growth analyses showed that follicle growth was significantly improved when hMSCs were present as support cells in 2D or 3D compared to follicles cultured alone (Figure 7B; Figure S3). Therefore, follicles reached significantly larger sizes with hMSCs in 2D (188.0 ± 52.4 µm) and with hMSCs in 3D (171.5 ± 47.9 µm) as compared to follicle only controls (146.7 ± 34.8 µm). Longitudinal evaluations revealed that follicles cultured alone grew almost three times more within the last two days of culture compared to the first three days. Follicles cultured with hMSC in 2D and 3D already grew more from the beginning of culture (Table S3). For 2D co‐cultures with hMSC a continuous increased growth was observed, while for 3D co‐cultures the follicle growth seemed to be transiently restricted between day three and five of culture. Analysis of GC proliferation by incorporation of 5‐bromo‐2’‐deoxyuridine (BrdU) during days 3 to 7 of culture did not show obvious differences between follicle cultures without and with hMSCs (Figure S4). However, oocytes of follicles cultured with hMSC during seven days grew significantly more than of follicles, cultured without (Figure 7C). Oocytes of follicles, which were cultured in direct contact with hMSC, showed a slight, but not significantly larger normalized oocyte size, compared to culture with hMSC in 2D.

**TABLE 2:** Numbers of encapsulated, surviving and growing follicles for follicles cultured alone, with hMSC in 2D on top or with hMSC in 3D. After seven days of culture follicles were identified as viable if there was a visible central round oocyte, intact and symmetrical appearing granulosa cells and an overall round follicle morphology detectable from brightfield images. Follicles were further classified as growing if they were viable and increased in diameter compared to day 0. Values for surviving and growing follicles are assessed from the combined pool of all follicles per condition. N = 5 independent experiments, and n = 16 ovaries.

7 days of culture (intermediate TG‐PEG‐RGD)	Follicles only	With hMSC in 2D	With hMSC in 3D
# Encapsulated follicles	67	60	75
# Surviving follicles	36 (54%)	26 (43%)	41 (55%)
# Growing follicles	32 (55%)	24 (40%)	41 (55%)

In our experiments, co‐cultures based on paracrine signaling and direct cell–cell contact both support follicle growth. The latter approach additionally allows to study cell–cell interactions in this model of the ovary in great detail, for example via immunofluorescent stainings. Stainings done for laminin and human fibronectin after seven days of culture showed that follicles and support cells both secrete extracellular matrix (ECM) into their surroundings (Figure 7D). Furthermore, follicles appeared embedded into the network of support cells and their spreading was not impaired by the follicles. Close‐up images further revealed that the support cells are in direct contact with the follicles and the follicle secreted laminin (Figure 7E).

## 3 Discussion

In this study, we have developed a TG‐PEG‐based follicle culture system that promotes growth of morphologically intact and functional murine follicles. By co‐culturing support cells with follicles, we showed the supportive function of their paracrine signals on follicle growth and the establishment of cell‐follicle interactions.

### 3.1 PEG Hydrogels are Suitable Substrates for Follicle Culture

The culture of medium to large size (100–180 µm start size) mouse follicles is well established, and several groups have shown success in growing them in 3D cultures using alginate, collagen or fibrin as scaffolds [16, 17, 33]. For most of these systems, the aim was to maximize follicle viability and functional growth to help develop methods that can potentially be translated to human to assist in fertility preservation. In our study, we successfully used our fully synthetic, enzymatically cross‐linkable TG‐PEG hydrogels to grow follicles in 3D culture. In these follicles, a continuous layer of laminin and fibronectin surrounding the GC layer together with the expression of Cx43 on GC, demonstrated the presence of a basal lamina and gap junctions between GC, key morphological characteristics of functional follicles [10, 11, 12, 13]. In vitro maturation further confirmed the suitability of TG‐PEG hydrogels for the culture of follicles, which promote mouse follicle development and enable the acquisition of oocyte competence similar to previous studies [22, 34]. This marks an essential step in replicating folliculogenesis in vitro, as follicles must not only grow but also function properly [35] for the culture system to aid in fertility preservation.

### 3.2 Influence of Mechanical Properties on Follicle Growth

We optimized the mechanical and biological properties of TG‐PEG hydrogels to match the requirements of follicles from different stages of development and to enable follicle growth using hydrogels with low (50 Pa), intermediate (400 Pa), and high stiffness (600 Pa). We found that encapsulated larger follicles grow better in soft TG‐PEG scaffolds, whereas the stiffness does not matter significantly for smaller follicles. A study showed that follicles of 100–130 µm and 150–180 µm grow best in alginate gels of approx. 200 Pa and less in hydrogels of 1300 Pa and more [36]. Similarly, it was demonstrated that follicles of start size of 100–120 µm grow well in TG‐PEG hydrogels up to 1000 Pa and growth rates decrease in hydrogels with higher stiffness [22]. When follicles grow, they either degrade or displace hydrogel components from their immediate surroundings, which likely has strongest implications on the largest follicles, as these have, besides a growing oocyte, a higher number of granulosa cells that could potentially proliferate. Governed by the defined properties of all hydrogel building blocks, the chosen stiffness of the TG‐PEG hydrogels could be kept stable throughout the full culture period. This adaptability and easy control over mechanical properties is clearly an advantage of TG‐PEG hydrogels when compared to the naturally derived alginate [20], that depending on alginate monomers and the concentration of divalent ions in the surrounding media change their mechanical properties [21]. Additionally, PEG hydrogels, could allow the building in of previously described modules that are degradable in a tightly controlled spatial, temporal, or trigger‐controlled manner [37, 38, 39] enabling a precise adaptation of the hydrogel in its supportive role during culture. This dynamic flexibility of hydrogel properties could help to more precisely and interactively control culture conditions for follicles of different developmental stages, especially for small human follicles that need to undergo significant growth.

### 3.3 Cell Adhesion Ligands Support Follicle Growth

To build a mechanically tunable synthetic culture model, which also promotes the growth of follicle support cells, we incorporated the cell adhesion ligand RGD into our hydrogel. The RGD concentration we used to form the TG‐PEG‐RGD hydrogels was based on our previous work, where the effect of different RGD concentrations in the PEG hydrogels was evaluated using human umbilical vein endothelial cells [28].

We found an increased follicle growth when TG‐PEG hydrogels were modified with RGD. This is in agreement with earlier studies demonstrating that the addition of RGD into the otherwise bioinert alginate hydrogels promotes the growth of two‐layered secondary mouse follicles and the acquisition of meiotic competence of oocytes from multilayered follicles [33]. Tomaszewski et al. demonstrated that mouse follicle growth is improved in bioactive PEG‐formulations compared to a bioinert control [23]. Given the presence of a basement membrane surrounding the follicle, this seems counterintuitive, as GCs are encapsulated by the basal lamina and their access to RGD may be restricted. This rather suggests that theca cells remained attached to the isolated follicles and may benefit from the cell adhesion sites of the hydrogel. Therefore, these findings highlight the importance of designing follicle culture substrates such that in addition to mechanical support, they also replicate key biological aspects of the ovarian tissue.

### 3.4 Follicle and Oocyte Growth Support via Paracrine Signaling

In our cocultures, the follicles showed no improved viability due to paracrine signals from mouse ovarian stromal cells (mOSCs), but there was a general trend towards enhanced growth, especially in follicles with a diameter of over 120 µm. Similarly, human mesenchymal stromal cells (hMSCs), which are known for their strong paracrine activity, did not increase viability and proliferation of GC but did improve follicle growth. Interestingly, both supporting cells used in the coculture system also resulted in enlarged oocytes, indicating functional follicle growth. These findings partially corroborate and complement previous reports where the 2D culture of ovarian cells led to improved growth and increased viability of follicles in the vicinity of follicle‐containing hydrogels [16, 24, 25, 26]. Furthermore, these observations suggest that these are general effects that are relatively independent of the cell type used for follicle co‐culture.

The growth and maturation of follicles and oocytes in the ovarian microenvironment are likely regulated by a mixture of paracrine and juxtacrine signals released by one or more specific cell types [8, 9]. Therefore, it is surprising that the mouse ovarian stromal cells used here provide only minor support for the follicle culture. One reason for this could be that both the isolation method and the culture medium have led to the selection of cell types that emit little paracrine signaling. We cannot rule out that follicle viability may depend on the high density of supporting cells, which could deplete significant amounts of nutrients from the provided culture medium, thereby creating suboptimal growth conditions.

### 3.5 Formation of an Engineered Ovary Model

This work was based on the hypothesis that engineered ovarian stroma tissues optimally support in vitro growth of functional follicles. An earlier study showed that the growth of collagen‐encapsulated follicles was improved when they were brought in contact with a sheet of interstitial ovary cells as compared to controls with granulosa cells or without cells [16]. Additionally, a protocol for the co‐encapsulation of mouse follicles and mouse embryonic fibroblasts in chemically crosslinked PEG hydrogels highlighted the importance of support cells for follicle growth and survival [40]. Nonetheless, the proposed concentrations of embryonic fibroblasts were significantly lower than in the ovary, which is why we used higher cell concentrations.

We have previously shown that TG‐PEG hydrogels containing a proteolytically degradable backbone and the cell adhesion ligand RGD support the formation of hMSC‐based stromal tissue. In this setup hMSC deposited their own ECM and generated their own cell‐specific microenvironment [32]. Importantly, when used for the 3D co‐culture of follicles here, hMSC additionally to the deposition of ECM molecules established direct contacts, enabling juxtacrine signaling with the growing follicles. Such cultures more closely mimic the native microenvironment of the ovary compared to previous cultures based solely on paracrine effects. However, while in our study, direct co‐cultures showed comparable growth of follicles and oocytes to paracrine signaling by support cells, the pattern of follicle growth was different. Follicles in indirect co‐culture grew more toward the end of the culture period, and the ones in direct co‐culture started earlier.

Furthermore, comparable follicle growth between indirect and direct co‐culture in our experiments could be attributed to the fact that co‐cultures of murine follicles were performed with human MSC. The direct co‐culture using hMSC presents a promising starting point for the testing and identification of physiologically more relevant support cells coming from the ovary. Once identified the hydrogel will need to be tailored to the needs of the specific murine ovary stromal cell populations, since (unpublished preliminary data) they did not show the typical stromal cell growth behavior in 3D.

Together, using our TG‐PEG‐based follicle culture platform, we present a straightforward approach to characterize and engineer cell–cell and cell‐matrix interactions between follicles, support cells, and their secreted ECM. The spatial arrangement of cells at high densities, follicles, and ECM, as established in our direct co‐culture, is an important step toward recapitulating the natural ovary in a meaningful way and to provide a platform to support functional follicle development. Besides enabling follicle growth, our culture model allows to detect and study cell–cell and cell‐matrix interactions in great detail. Additionally, the effect of culture conditions, new compounds, and materials on follicle culture can be directly assessed. Therefore, when translated to humans, new drugs which affect the ovary could be tested in this newly engineered ovarian model.

## 4 Conclusion

In this work, we successfully developed a mouse follicle culture system to promote follicle growth and to study crucial stromal cell‐follicle interactions. By assessing the effect of TG‐PEG stiffness and presence of the cell adhesion peptide RGD, we were able to demonstrate the importance of the mechanical properties of the scaffold, depending on developmental stages of follicles, as well as the importance of incorporating ECM derived cues. The modular, synthetic TG‐PEG used to grow follicles was shown to accommodate follicle growth, and preserve key morphological characteristics, like basal lamina proteins and connexin 43, a gap junction protein needed for physiological follicle growth. Our study, furthermore, highlights that ovarian stroma cells play a crucial role in follicle development and are needed to further improve follicle growth. The established direct co‐culture of cells at high concentrations and follicles in TG‐PEG‐RGD additionally provides an excellent way of detecting and visualizing cell–cell interactions. In the future, the tunability of TG‐PEG hydrogels will enable an even more precise adaption of the material in its supportive role, which will be of the highest interest especially for the growth of small human follicles.

## 5 Methods

### 5.1 Ethics

Animal care and housing was performed according to protocols approved by the Veterinary Office of Zurich, Switzerland (license number ZH163/2024). Water and food was provided ad libitum and a total of 26 female mice at the age of 14–28 days were euthanized for the experiments. Human mesenchymal stromal cells (hMSC) were extracted from bone marrow aspirates taken from healthy patients undergoing hip surgery at the Univeritätsspital Basel after informed consent (Prof. Kummer; approval date 26/03/2007 Ref. Number 78/07).

### 5.2 Synthesis and Fabrication of TG‐PEG Hydrogels

Transglutaminase crosslinked PEG (TG‐PEG) was synthesized as previously described [28, 29, 32]. In brief, 8‐arm PEG‐vinylsulfone (40 kDa) was functionalized with one of two substrates of transglutaminase factor XIII (FXIII), a glutamine acceptor peptide (Gln; H‐NQEVSPL‐ERCG‐NH2, Bachem) or with a lysine donor peptide, which contains an MMP‐degradable sequence (MMP‐sensitive Lys; Ac‐FKGG‐GPQGIWGQ‐ERCG‐NH2, Bachem). The functionalization was performed for 2 h at 37°C and pH 8. The resulting PEG‐Gln and PEG‐MMP‐Lys were mixed together in a 1:1 molar ratio in Tris buffer (50 mm, pH 7.6) with 50 mm calcium chloride. Upon addition of 10 U ml^−1^ activated FXIII, a hydrogel formed within approximately 3–4 min. For experiments including a cell adhesion ligand 50 µm H‐FKGGRGDSPG‐NH2 (Lys‐RGD, Bachem) was added to the TG‐PEG monomer mix before addition of FXIII.

### 5.3 Rheological Characterization of TG‐PEG Hydrogels

TG‐PEG hydrogels were mechanically characterized by measuring the storage modulus G’ (corresponding to stiffness) with an oscillatory strain test using a MCR 302e rheometer from Anton Paar. Data was collected using an 8 mm diameter parallel measuring plate, together with a sample stage heated to 37°C. Freshly prepared hydrogels with different initial TG‐PEG concentrations were analyzed (in situ analysis) over 30 min at a strain amplitude of 4% and an angular frequency of 1 rad s^−1^, conditions, which were previously determined to fall into the linear viscoelastic region. The stiffness of the different PEG concentrations was determined by taking the G’ of each gelation curve after 30 min.

To determine the TG‐PEG hydrogel stiffness during seven days of culture, disc shaped TG‐PEG hydrogels of 60 µl were prepared using two microscope slides and spacers in a sandwiching method. To match the stiffness of the sandwich hydrogel with the corresponding in situ hydrogel, the measuring height of the rheometer was adapted so that the measured G’ of the sandwich hydrogel fits the G’ of the corresponding in situ characterized TG‐PEG hydrogel. This measuring height was determined once for each gel individually and then used throughout seven days of measurement. For the duration of the experiment, the hydrogels were stored in follicle culture medium at 37°C with 5% CO_2_ in a humidified chamber and medium was changed every second day.

### 5.4 Isolation of Mouse Follicles

Follicles were isolated from 14–28 days old C57BL/6j mice using a mechanical approach. After euthanasia of the mice by CO_2_ exposure followed by induction of a pneumothorax, the ovaries were explanted and stored in L15‐Leibowitz medium (Gibco) supplemented with 5% fetal bovine serum (FBS, Cytiva) and stored on ice until further usage. Two insulin gauge needles were used to first clean the ovaries by removing the bursa and fat tissue and afterward to flick individual follicles out of the ovary. For this, ovaries were individually placed in a dish containing warm L15‐Leibowitz medium with 1% FBS and 0.5% penicillin/streptomycin (PS, 10 U mL^−1^ penicillin, 10 µg mL^−1^ streptomycin, Gibco) and were then immobilized by pinning the ovary down onto the bottom of the dish with one needle while using the other needle to apply rapid strokes to create vibration in the needle pinning down the ovary. Using this vibration‐based technique, individual follicles were released into the dish. This was repeated until the ovary was completely disintegrated. Then, follicles were collected and stored in Minimal Essential Medium alpha with Glutamax (aMEM Glutamax based medium containing 1% FBS and 0.5% PS) in a U‐shaped ultra‐low adhesion wellplate to prevent sticking of cells to the wells (costar). The whole process of follicle release and collection was limited to 40 min per ovary to minimize stress due to changes in temperature and pH conditions, and the collected follicles were stored at 37°C with 5% CO_2_ in a humidified incubator as soon as possible to keep temperature and pH changes minimal. A total of 32 female mice were sacrificed for this study and the exact number of ovaries used for each experiment is stated in the respective figure and table caption.

### 5.5 Encapsulation and Culture of Follicles

TG‐PEG hydrogel mixes were prepared according to the number of healthy‐looking isolated follicles. A follicle was counted as healthy when the oocyte was round and visible in the center of the follicle and the overall shape of the follicle was round with no granulosa cells appearing damaged. Per ten follicles one hydrogel of 8 µl was prepared. Hydrogel mixes were prepared by mixing all components but the follicles in medium together. In parallel, follicles were collected from the wellplate and spun down at 80 rcf for 5 min. Next, the pelleted follicles were collected in the desired amount of medium and added to the hydrogel mix. After the addition of FXIII and mixing of the solution, 8 µl hydrogels were dispensed into wells of a flat‐bottom 96‐wellplate with care taken to place the gel centrally in the well. As the follicles sediment fast in the hydrogel mix, it was pipetted up and down three times before each new hydrogel was pipetted, and a maximum of six hydrogels were pipetted at once, to ensure that gelation had not yet started when all gels are pipetted. To distribute the follicles evenly throughout the hydrogels, the well plate was carefully inverted every 20 s for at least six minutes after the addition of FXIII. Eight minutes after initiation of gelation 150 µl of pre‐gased base growth medium (GM ‐/‐, consisting of aMEM GlutaMAX with 3 mg ml^−1^ bovine serum albumin (BSA, AppliChem), 1 mg ml^−1^ fetuin from fetal bovine serum (Sigma–Aldrich)) were carefully added to each well and the plate was stored in the incubator. After 30 min, medium was replaced with full growth medium (GM +/+, consisting of pre‐gased GM ‐/‐ with 0.1% insulin‐transferrin‐selenium (ITS Premix, Corning) and 10 mIU ml^−1^ follicle stimulating hormone (FSH from human pituitary, Sigma–Aldrich)), and during culture time, half of the medium was replaced every two days with freshly prepared GM +/+. For TG‐PEG stiffness and co‐culture experiments at least 50 healthy looking follicles were encapsulated per experimental group, for analysis of the effect of RGD adhesion peptide at least 25 follicles per condition.

### 5.6 Cells Used for Co‐Culture and Preparation of Hydrogels

For the co‐culture of follicles and support cells, mouse ovary stroma cells (mOSC) or human mesenchymal stromal cells (hMSC) were used. hMSC were extracted from bone marrow aspirates and mOSC were obtained by plating the pieces of ovary tissue which remain after follicle isolation into cell culture flasks. After several days, migratory cells attached to the flask and cells could be passaged and expanded. Both cell types were cultured in aMEM medium containing 10% FBS, and 1% PS. Medium of hMSC additionally contained 5 ng ml^−1^ basic fibroblast growth factor (FGF‐2, Peprotech). Cells were used between passage 3–4 (mOSC) and passage 4–7 (hMSC) and they were detached when 90% confluent using 0.05% Trypsin‐EDTA (Gibco).

Before using the hMSC for co‐culture they were stained with Cell Trace Far Red (Invitrogen) to facilitate detection of human cells during and after culture. Cells were resuspended at 20 Mio cells ml^−1^ in medium and then the desired number of cells was diluted in phosphate buffered saline (PBS, Gibco). 1 million cells to be stained were diluted in 1 mL PBS and 1 µl of Cell Trace staining solution. Cells were then incubated for 20 min in the dark at room temperature (RT), before 5 mL of medium containing FBS was added to incubate another 5 min. Then, cells were pelleted and resuspended in medium at the desired concentration. For the co‐culture condition where support cells are in 2D, 8000 cells were added to each hydrogel with the medium changed 30 min after initiation of gelation. For the co‐culture condition where cells and follicles are embedded together within the hydrogel, cells were added to the hydrogel mix to have a final concentration of 3 Mio cells ml^−1^. Experiments with mOSC only included the condition where they are afterward seeded in 2D on top of the hydrogel.

### 5.7 Analysis of Follicle Growth

To analyze how follicle size develops during culture, brightfield images of each follicle were taken on day 0, 3, 5, and 7 after encapsulation using a LEICA Inverted DMI6000B fluorescence microscope. From the images, two perpendicular diameters were measured per follicle using imageJ and the average was calculated. After seven days of culture follicles were identified viable if there was a visible central round oocyte, intact and symmetrical appearing granulosa cells and an overall round follicle morphology detectable from brightfield images. Viable follicles further classified into growing and not growing, according to the size increase from day 0 to day 7. Number of viable and growing follicles were separately stated in tables, and only growing follicles were used to plot growth. Mean size of growing follicles per condition and timepoint was used to calculate the slope of mean follicle growth (in µm).

### 5.8 In Vitro Maturation (IVM)

After 8 days of culture follicles were retrieved from the hydrogels by digesting each TG‐PEG‐RGD hydrogel with 100 µl of 2 mg ml^−1^ collagenase A solution at 37°C. As soon as the TG‐PEG‐RGD liquefied, 100 µl of cold media supplemented with FBS were added per well to deactivate the collagenase and prevent follicle digestion. Follicles were picked and transferred into fresh medium to remove all traces of the enzyme. Next, they were transferred into maturation medium (GM ‐/‐ containing 5 ng ml^−1^ epithelial growth factor (EGF, Peprotech) and 1.5 IU ml^−1^ human chorionic gonadotropin (hCG, Sigma–Aldrich)) and incubated at 37°C and 5% CO_2_ in a humidified chamber for 16 h. Care was taken that the follicles did not come in direct contact with each other as this would lead to the formation of a cell‐aggregate. To remove the somatic cells from the oocyte after incubation in maturation medium, follicles were treated with 0.2% hyaluronidase (Sigma–Aldrich) in GM‐/‐ for 30 min followed by stripping of the cells using a small‐bore pipet. For the last 20 min of hyaluronidase incubation, 2 mm Hoechst 33342 solution (Thermo Scientific) was added 1:250 to the hyaluronidase solution to stain the DNA. Oocytes were finally imaged directly using a LEICA Inverted DMI6000B fluorescence microscope without further washing.

### 5.9 Immunofluorescence Staining of Follicles and Confocal Laser‐Scanning Microscopy (CLSM)

In TG‐PEG hydrogels embedded follicles were washed two times 3 min with warm PBS to remove any medium. Afterward, they were fixed for 30 min at RT in 4% paraformaldehyde (PFA, Artechemis), followed by permeabilization and blocking for 30 min at RT shaking in 1% BSA/PBS with 0.3% Triton x‐100 (Sigma–Aldrich). Samples were incubated in 1 % BSA/PBS with 0.1% Triton x‐100 and primary antibodies at 4°C shaking overnight. Primary antibodies for laminin (ab7463, Abcam) and fibronectin (sc59826 used for hMSC, Santa Cruz; ab2413 used for mouse follicles, Abcam) were used at 1:200 and primary antibody for Connexin 43 (ab11370, Abcam) was used at 1:100. Next, samples were washed for at least 3 h at RT in PBS before incubating in 1% BSA/PBS with the secondary antibodies (ab150077 from Abcam, A 21037 from Thermo Fisher) 4′,6‐diamidino‐2‐phenylindole (DAPI, Sigma–Aldrich) and Phalloidine‐Rhodamine (Invitrogen, R415) at 4°C degrees shaking overnight. Secondary antibodies were used at 1:200, DAPI at 1:10’000 and Phalloidine‐Rhodamine at 1:4000.

Confocal images were acquired with an inverted laser‐scanning microscope (Leica STELLARIS 5) and images were analyzed and processed in imageJ. Single plane images depicting individual stainings, or composites, and maximum projections of acquired stacks with respective step size of imaging are shown as indicated in the figure captions.

### 5.10 Analysis of Follicle Development

To assess the functionality of follicle growth, oocyte sizes and proliferation of granulosa cells were analyzed. Oocyte sizes were measured from brightfield images taken on day 0 and day 7 after encapsulation of follicles cultured alone in TG‐PEG‐RGD or with mOSC or hMSC in co‐cultures. Viable and growing follicles where the oocyte was clearly visible, were analyzed. Normalized oocyte growth was plotted, by dividing the size on day 7 with the size of the same oocyte at day 0. Granulosa cell proliferation was analyzed using a 5‐bromo‐2’‐deoxyuridine (BrdU) labeling and detection protocol. A 10 mm BrdU stock solution was prepared by dissolving 3 mg BrdU (ab142567, abcam) in 1 mL water. This stock solution was diluted 1:500 in fresh follicle medium before it was sterile filtered, and ITS and FSH were added. BrdU‐supplemented full follicle growth medium was added to the follicle cultures on day 3 and day 5 after encapsulation to have a final concentration of 1:000. At day 3 of culture, half and at day 5 the whole medium was replaced by fresh BrdU‐supplemented follicle medium. After seven days of culture, hydrogels were washed two times 3 min with warm PBS and then fixed for 30 min at RT in 4% paraformaldehyde (PFA, Artechemis). Samples were then permeabilized and blocked for 30 min at RT shaking in 1% BSA/PBS with 0.3 % Triton x‐100 (Sigma–Aldrich) before DNA was hydrolyzed by incubation in 1 m HCl at RT. Incubation with the primary antibody for BrdU (ab6326, Abcam) diluted 1:200 in 1% BSA/PBS with 0.1% Triton x‐100 was carried out at 4°C shaking overnight. After at least 3 h of washing in PBS at RT, samples were incubated overnight at 4°C with the secondary antibody (ab1500166, Abcam), 4′,6‐diamidino‐2‐phenylindole (DAPI, Sigma–Aldrich, used at 1:100) and phalloidin (Phalloidin‐633, Invitrogen, used at 1:400) diluted in 1% BSA/PBS with 0.1% Triton x‐100.

Confocal images were acquired with an inverted laser‐scanning microscope (Leica STELLARIS 5) and images were analyzed and processed in imageJ.

### 5.11 Statistical Analysis

Statistical analysis of follicle growth data was performed using GraphPad Prism. T‐Test or one‐way ANOVA were used as stated in the figure captions. A *p*‐value < 0.05 was considered statistically significant. *, **, ***, **** were used in the figures to denote *p*<0.05, 0.01, 0.001, 0.0001 respectively. All data are reported as mean ± standard deviation.

## Funding

Southeast Asia – Europe Joint Funding Scheme 2020 through Swiss National Science Foundation (IZJFZ3_202474).

## Conflicts of Interest

The authors declare no conflicts of interest.

## Supporting information




**Supporting File**: adhm70958‐sup‐0001‐SuppMat.docx.

## Data Availability

The data that support the findings of this study are available from the corresponding author upon reasonable request.
