# Supplementary material for: Three‐Dimensional Ovary Model to Improve and Study Murine Follicle Growth
Source: Adv Healthc Mater. 2026 Feb 24;15(17):e04643. doi: 10.1002/adhm.202504643 (PMC13175289; doi:10.1002/adhm.202504643)
Supplement: Supplementary file 1 — Supporting File: adhm70958‐sup‐0001‐SuppMat.docx. [file ADHM-15-0-s001.docx]

Supporting Information

Three-dimensional Ovary Model to Improve and Study Murine Follicle Growth

Mira Jacobs, Valon Gllareva, Lukas Moser, Silvia Pravato, Eric Mora Pimentel, Brigitte Leeners, Martin Ehrbar*


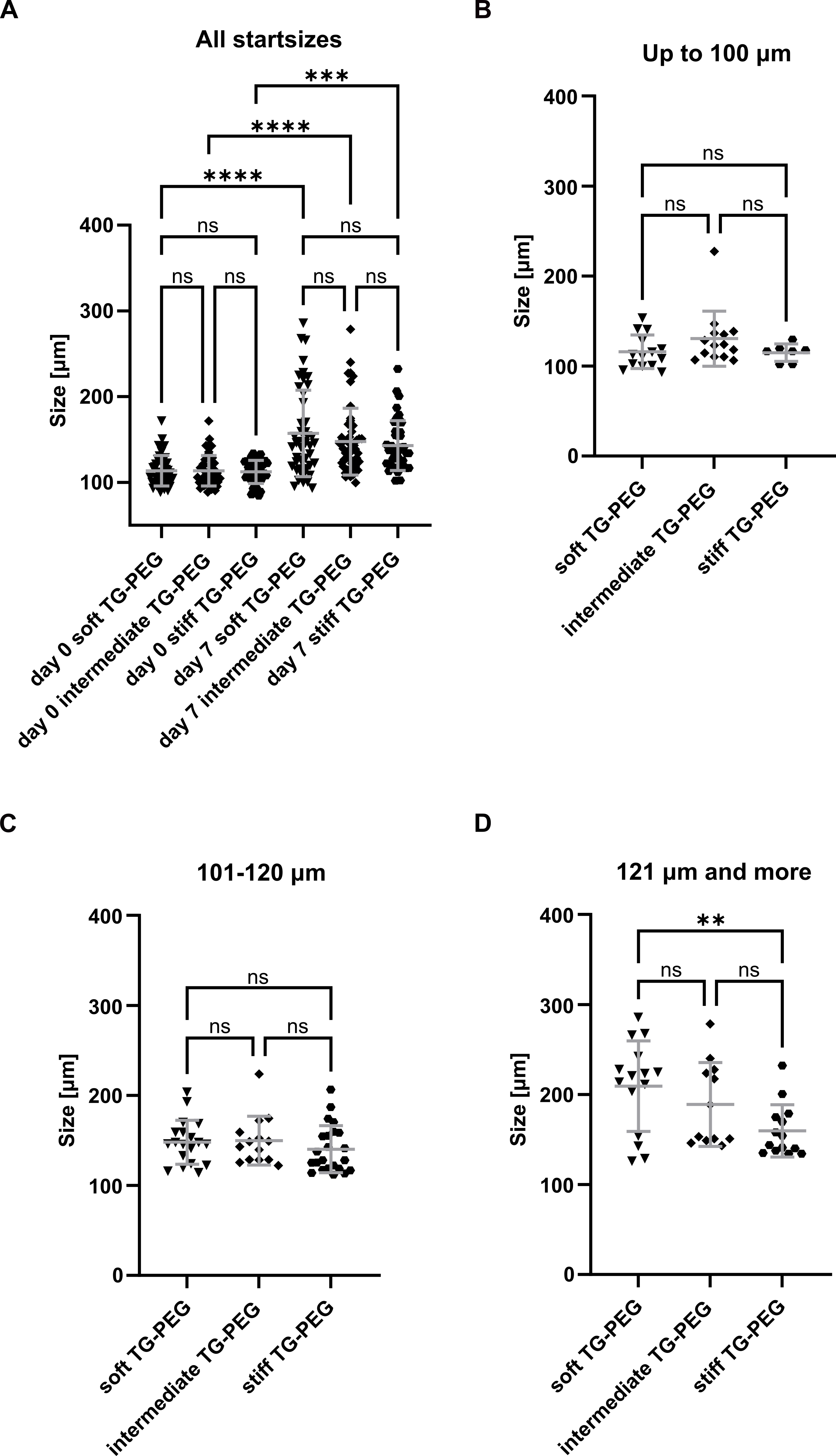


**Figure S1.** Statistical analysis of follicles cultured in TG-PEG of different stiffnesses. Size of live follicles embedded in soft, intermediate and stiff TG-PEG. Data of all growing follicles per condition after encapsulation and after seven days of culture is plotted (A), and for the different start sizes after seven days of culture. Follicles up to 100 µm size (B), 101-120 µm (C), and more than 120 µm (D). One-way ANOVA with multiple comparisons used to determine statistical significances between groups (**p<0.01, ***p<0.001, ****p<0.0001).

**Table S1.** Numbers of encapsulated, surviving and growing follicles for intermediate TG-PEG hydrogels modified with RGD or without. After seven days of culture follicles were identified viable if there was a visible centrally located round oocyte, intact and symmetrical appearing granulosa cells and an overall round follicle morphology detectable from brightfield images. Follicles were classified further as growing, if they were viable and additionally increased in diameter compared to day 0. Data are from N= 3 independent experiments, and n= 8 ovaries.

| **7 days of culture (1.7% TG-PEG)** | **With RGD** | **Without RGD** |
| --- | --- | --- |
| # Encapsulated follicles | 59 | 36 |
| # Surviving follicles | 45 (76%) | 22 (61%) |
| # Growing follicles | 33 (56%) | 18 (50%) |


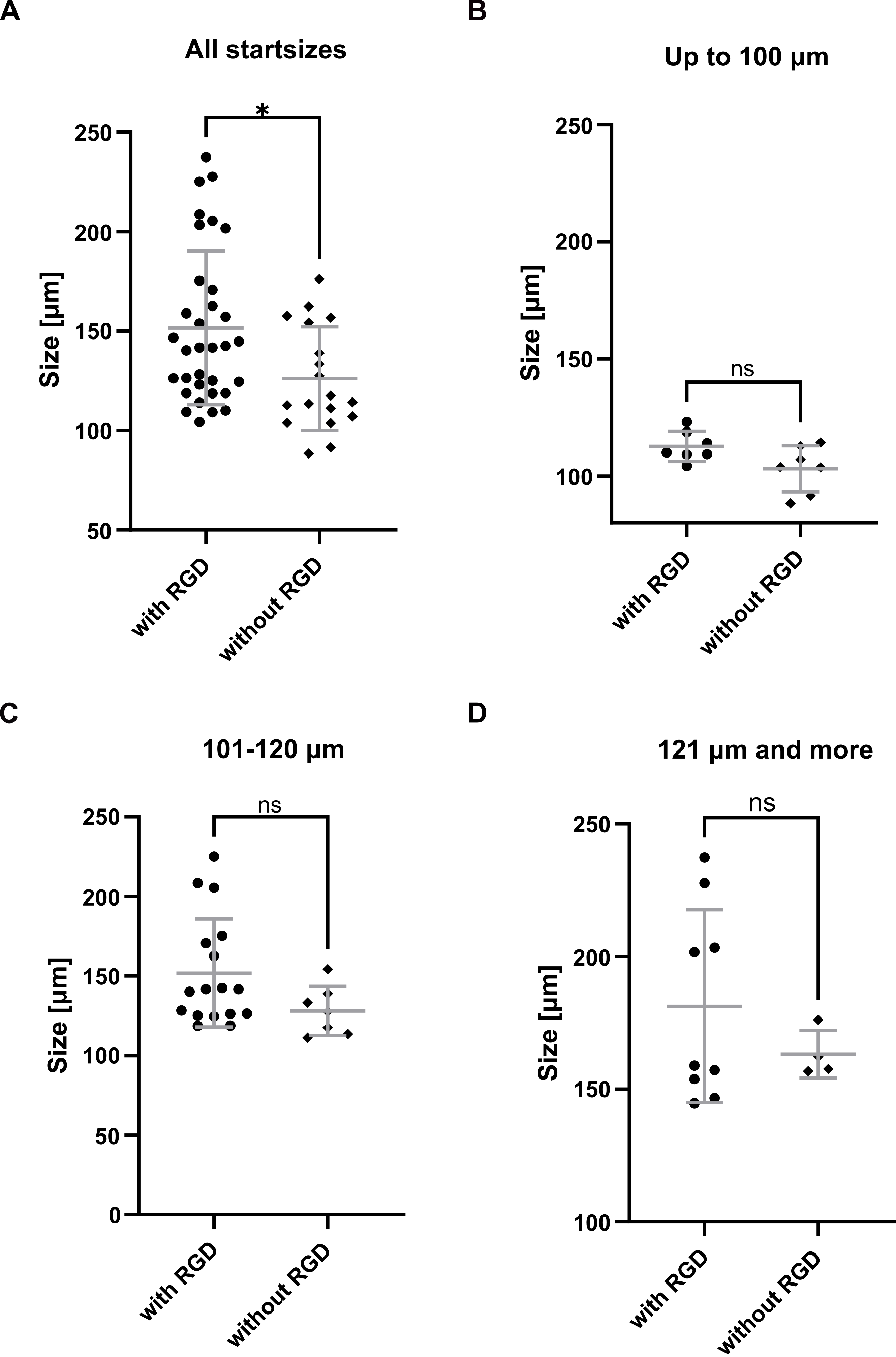


**Figure S2.** Statistical analysis of follicles cultured in intermediate TG-PEG modified with RGD or without. Size of all live follicles embedded in TG-PEG with or without RGD (A), and of the different start sizes after seven days of culture. Follicles up to 100 µm size (B), 101-120 µm (C), and more than 120 µm (D). Unpaired t-test used to determine statistical significances between conditions (*p<0.05).

**Table S2.** Numbers of encapsulated, surviving and growing follicles in intermediate TG-PEG-RGD hydrogels cultured alone or with mOSC on top in 2D. After seven days of culture follicles were identified viable if there was a visible centrally located round oocyte, intact and symmetrical appearing granulosa cells and an overall round follicle morphology detectable from brightfield images. Follicles were classified further as growing, if they were viable and additionally increased in diameter compared to day 0. N= 2 independent experiments, and n= 8 ovaries.

| **7 days of culture (intermediate TG-PEG-RGD)** | **Follicles only** | **With mOSC on top in 2D** |
| --- | --- | --- |
| # Encapsulated follicles | 61 | 94 |
| # Surviving follicles | 42 (69%) | 62 (66%) |
| # Growing follicles | 39 (64%) | 56 (60%) |


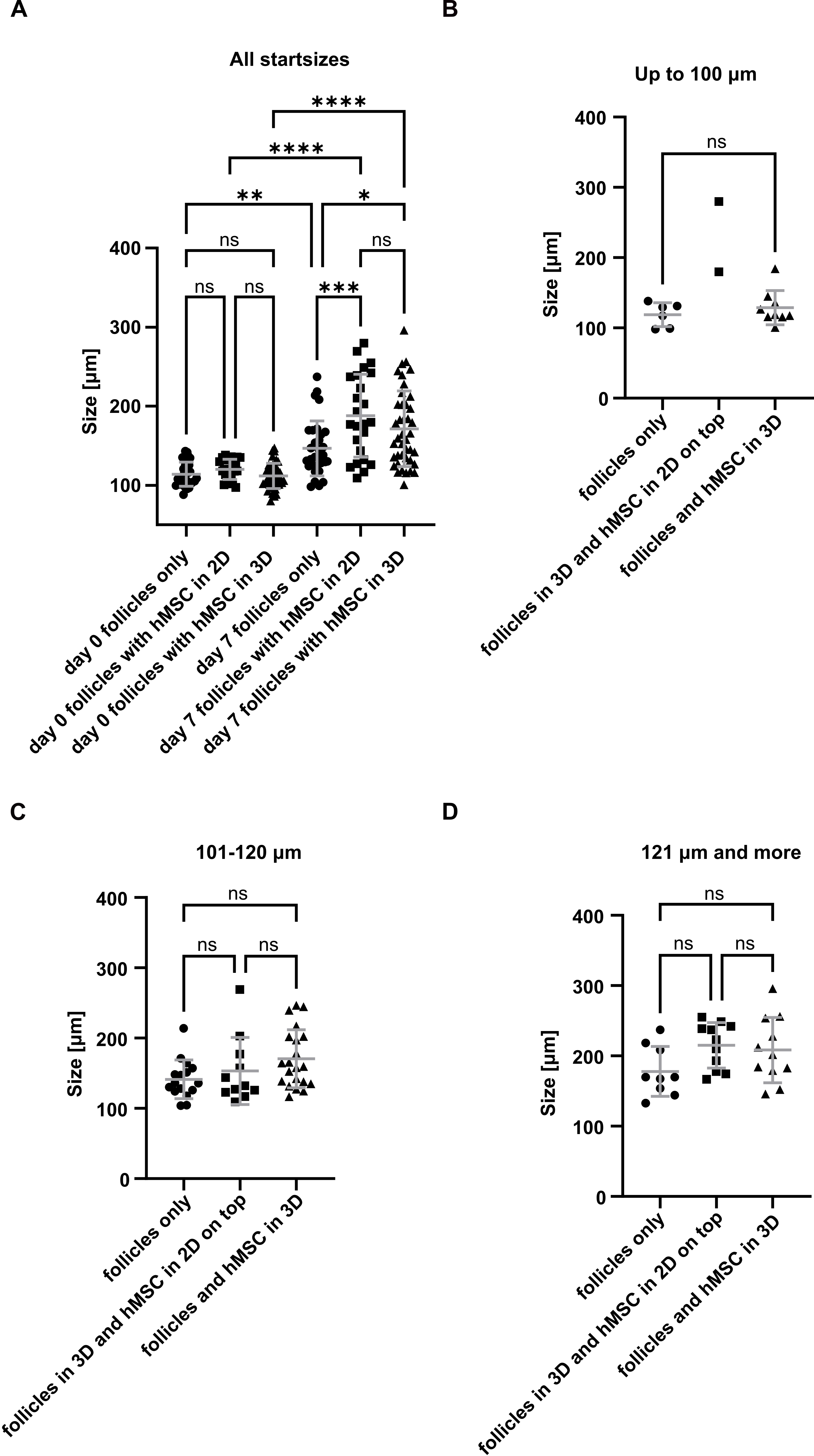


**Figure S3.** Statistical analysis of follicles cultured in intermediate TG-PEG-RGD alone, or with hMSC as support cells. Size of live follicles embedded in soft, intermediate and stiff TG-PEG. Data of all growing follicles per condition after encapsulation and after seven days of culture is plotted (A), and for the different start sizes after seven days of culture. Follicles up to 100 µm size (B), 101-120 µm (C), and more than 120 µm (D). One-way ANOVA with multiple comparisons used to determine statistical significances between groups (*p<0.5, **p<0.01, ***p<0.001, ****p<0.0001).

**Table S3.** Slope of mean follicle growth for different time intervals and culture conditions. The mean follicle sizes per condition and timepoint were used to calculate the slope of follicle growth between timepoints of size measurement. A larger value indicates a larger growth during this time. Data of growing follicles from all start sizes of the assessment of RGD effect and co-culture experiments with mOSC or hMSC were analyzed. The slope is presented in size increase (µm) per day for the given interval of culture.

|  | **Effect RGD** | | **Effect mOSC** | | **Effect hMSC** | | |
| --- | --- | --- | --- | --- | --- | --- | --- |
| In [µm/day] | **No RGD** | **+ RGD** | **Foll only** | **+ mOSC** | **Foll only** | **+ hMSC 2D** | **+ hMSC 3D** |
| # Day 0-3 | 1.1 | 2.3 | 1.4 | 3.7 | 1.9 | 6.8 | 10.6 |
| # Day 3-5 | 3.4 | 6.0 | 5.1 | 8.8 | 6.5 | 11.5 | 4.4 |
| # Day 5-7 | 4.3 | 10.4 | 8.1 | 11.4 | 7.0 | 12.2 | 9.6 |


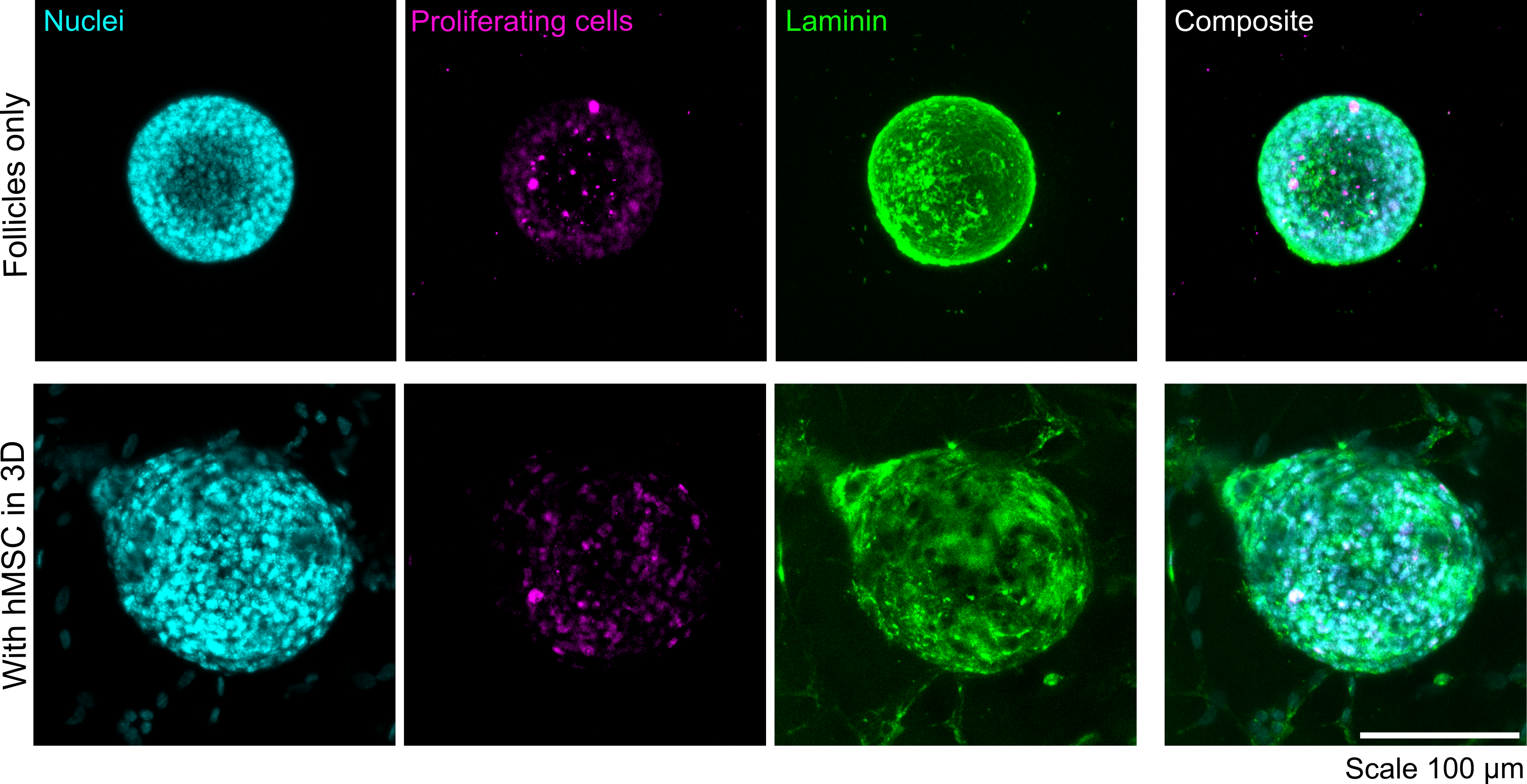


**Figure S4.** Staining of proliferating granulosa cells in co-culture experiments with hMSC. A BrdU-assay was performed to detect proliferating cells within follicles cultured in different conditions. Maximum projections of confocal stacks (step size 3 μm) shown for follicles cultured either alone or with hMSC in 3D for seven days. N= 2 experimental days, and n= 8 ovaries. Samples stained with DAPI (in cyan), and with specific antibodies for BrdU (in magenta), and laminin (in green), to detect cell nuclei, proliferating cells, and the basal lamina of follicles, respectively. Scale = 100 μm.
